# Unseen suffering: the urgent need for gender-affirming pain and mental health management for transgender individuals in India

**DOI:** 10.3389/fpubh.2025.1594703

**Published:** 2025-04-29

**Authors:** Anithamol Babu

**Affiliations:** ^1^School of Social Work, Marian College Kuttikkanam Autonomous, Kuttikkanam, India; ^2^School of Social Work, Tata Institute of Social Sciences Guwahati Off-Campus, Jalukbari, India

**Keywords:** transgender healthcare, pain management, mental health disparities, AI-driven interventions, gender-affirming care

The absence of a structured, evidence-based, and gender-affirming framework for pain management among transgender individuals in India represents a profound failure of medical and psychiatric research, perpetuating systemic health disparities and reinforcing institutional neglect (1). Despite advancements in gender-affirming healthcare worldwide, transgender individuals in India continue to face compounded physical, psychological, and emotional distress due to inadequate pain management frameworks. While existing studies predominantly focus on transgender women's vulnerabilities, the healthcare needs of transgender men, particularly pain perception and mental health, remain understudied (2). Although some Indian states, such as Kerala, have pioneered gender clinics, Delhi has launched mental health initiatives for transgender individuals, Maharashtra has introduced targeted healthcare programs, and Tamil Nadu has implemented welfare policies for transgender persons, these efforts remain isolated and insufficient to address the widespread disparities in healthcare access (3–5). The lack of a cohesive national strategy results in inconsistent availability and quality of gender-affirming medical care across the country. The absence of a nationwide, standardized approach to transgender pain management contributes to fragmented care, uninformed clinical practices, non-standardized psychiatric interventions, and ultimately, suboptimal healthcare outcomes (6, 7). Without immediate academic inquiry and policy reform, transgender individuals will remain underserved in both medical and psychiatric settings, deprived of equitable and gender-affirming healthcare.

Hormone therapy, a fundamental aspect of medical transition, plays a critical role in shaping both pain perception and mental health outcomes (8). However, the long-term physiological and neurological effects of hormone replacement therapy (HRT) remain underexplored. Despite evidence suggesting that testosterone and estrogen therapies may influence pain modulation and psychiatric outcomes, there remains a lack of robust research on their long-term effects on chronic and neuropathic pain, warranting urgent investigation (8). The current absence of specialized treatment frameworks leads to generic, non-individualized pain management strategies, which fail to align with the specific neurobiological and psychological needs of transgender individuals.

The inadequacy of gender-affirming surgical pain management in India exemplifies a systemic failure to integrate evidence-based, gender-specific care into the healthcare framework, exacerbating the suffering of transgender individuals (5). Surgical interventions such as orchiectomy, vaginoplasty, mastectomy, phalloplasty, and metoidioplasty impose significant physiological and psychological burdens, yet the absence of specialized protocols results in a reliance on generic pain management strategies that disregard the unique needs of transgender patients (9). This neglect, compounded by the absence of structured psychiatric support, heightens vulnerability to trauma-related disorders and prolongs recovery (10). The well-documented interplay between physical pain and psychological distress is routinely ignored, as the healthcare system fails to acknowledge the compounding effects of gender dysphoria (currently known as experienced gender), systemic stigma, and social marginalization (11). The exclusion of gender-affirming psychiatric models from pain management frameworks further intensifies pain perception, reinforcing a cycle of suffering that remains unaddressed. A paradigm shift is imperative; comprehensive, multidisciplinary care must be embedded into transgender healthcare, integrating psychiatric interventions such as cognitive-behavioral therapy (CBT), trauma-informed approaches, and gender-affirming psychiatric models. CBT, a proven method for chronic pain management, targets maladaptive thought patterns, enhances coping mechanisms, and mitigates the psychological toll of persistent pain. Trauma-informed approaches are essential, recognizing the widespread medical trauma and systemic discrimination transgender individuals endure, ensuring patient autonomy, validating lived experiences, and fostering culturally competent, non-stigmatizing care. Without the systematic integration of psychiatric interventions into pain management, transgender individuals will continue to experience preventable suffering, fragmented care, and suboptimal health outcomes, reinforcing institutional neglect that contradicts the very principles of inclusive, patient-centered medicine (12).

The structural barriers within India's healthcare system further compound these challenges. Rooted in historical caste, gender, and communal hierarchies, these barriers reflect broader social determinants—such as housing insecurity, employment discrimination, and exclusion from insurance—that intensify pain, vulnerability and mental distress. Transgender individuals frequently encounter discrimination and inadequate medical knowledge among healthcare providers, leading to reluctance to seek medical attention (13). The absence of transgender-specific pain and mental health assessment tools, coupled with insufficient training in gender-affirming healthcare, results in inadequate and often inappropriate treatment (14). Addressing these systemic barriers requires targeted policy interventions, curriculum reform in medical education, and widespread implementation of gender-affirming clinical practices. Without these structural changes, transgender individuals will continue to receive suboptimal care, perpetuating cycles of medical neglect and exclusion (15, 16).

To rectify these deficiencies, a restructured approach to transgender pain and mental health management is essential. Critical research inquiries must be prioritized: how do hormone therapies differentially affect pain perception and psychiatric outcomes in transgender individuals? What specific pain and mental healthcare needs do transgender men have, and why has research historically neglected them? How can pain and psychiatric assessment tools be refined to incorporate the gendered and social dimensions of pain in transgender populations? What are the comparative outcomes of personalized, evidence-based pain and mental health interventions vs. the current generalized treatment protocols? How do intersectional factors, such as caste, socioeconomic status, geographic location, and disability, further influence transgender individuals' experiences of pain and mental health disparities? In particular, pain and mental health outcomes among transgender individuals are mediated by intersectional axes of marginalization, including caste-based discrimination, rural-urban healthcare gaps, age-specific vulnerabilities, class-linked barriers to private healthcare, and layered experiences of social exclusion. For instance, transgender individuals in rural areas often face compounded invisibility due to infrastructural deficiencies and stigma, while urban poor trans communities are constrained by exploitative care systems and must collectively inform future clinical and policy reform.

A rigorous, interdisciplinary research approach is required to effectively assess pain and mental health in transgender populations. A mixed-methods research design, incorporating both qualitative and quantitative methodologies, will provide comprehensive insights into pain experiences, psychiatric outcomes, and treatment efficacy. Qualitative methods such as in-depth interviews, focus groups, and participatory action research will allow for detailed explorations of lived experiences, while quantitative surveys and longitudinal studies will identify broader patterns in pain perception and mental health indicators. For example, Bhattacharya and Ghosh (7) document the lived experiences of pain and exclusion among Kolkata's hijra and kothi populations, revealing how community narratives can disrupt dominant biomedical interpretations. Similarly, Ranade et al. (1) used a mixed-method design in Mumbai, Delhi, and Bengaluru to unearth psycho-social pain narratives that remain largely silenced in mainstream psychiatric discourse. These studies underscore the value of narrative and ethnographic approaches in capturing the complexities of trans-specific pain and psychological distress, which are often obscured by conventional clinical metrics. Given the challenges of reaching transgender individuals within healthcare settings, a combination of respondent-driven sampling (RDS) and snowball sampling techniques should be employed to ensure inclusivity and representation. Collaborations with transgender-led organizations, healthcare institutions, and mental health advocacy groups will further facilitate ethical and community-driven research. Ethical research must extend beyond standard confidentiality measures to ensure meaningful participation and community engagement (17). Participatory ethics require transgender individuals to shape research priorities and co-develop objectives, while researchers must maintain reflexivity through practices such as journaling, peer debriefing, and participatory analysis. This ensures that transgender experiences are not merely studied but actively integrated into the development of healthcare interventions.

A fundamental transformation in transgender-inclusive healthcare is imperative. While India continues to grapple with systemic shortcomings in transgender healthcare, several countries have successfully implemented evidence-based, gender-affirming pain and mental health interventions. The Netherlands, for example, has developed comprehensive transgender healthcare frameworks integrating hormonal therapy, surgical interventions, and psychiatric support into a single continuum of care. The Dutch approach prioritizes early psychiatric evaluation and ensures that pain management is tailored to individual hormonal and surgical needs. Similarly, Canada and Australia have incorporated multidisciplinary gender clinics that address both physical and mental health concerns, reducing disparities in healthcare access. These countries emphasize trauma-informed psychiatric care, acknowledging the heightened vulnerability of transgender individuals to mental health disorders due to social stigma and medical neglect. Incorporating such best practices into India's healthcare system—particularly in terms of gender-affirming psychiatric models, pain management protocols, and medical education reforms—could significantly enhance transgender health outcomes.

Advancing AI-driven pain management models, integrating culturally competent psychiatric interventions, and adopting intersectional methodologies will be critical in addressing these deficiencies. The implementation of precision medicine, digital mental health tools, and gender-affirming clinical frameworks has the potential to revolutionize transgender healthcare in India. However, for these innovations to be effective, they must be accompanied by targeted medical education reforms and extensive healthcare provider training. Additionally, the integration of AI-driven healthcare solutions presents a promising avenue for improving transgender pain and mental health management in India. AI-based diagnostic tools and mental health chatbots—already in use in countries like the US and Germany—can improve assessments of pain and psychiatric needs while offering anonymous, immediate support for LGBTQ+ individuals (18, 19). These models could be adapted to the Indian context to bridge gaps in access to mental health services for transgender individuals facing discrimination in traditional healthcare settings. Moreover, telehealth platforms and mobile health (mHealth) applications have expanded access to gender-affirming psychiatric care. Digital health outreach programs in the UK, for instance, provide remote psychiatric counseling for transgender individuals, reducing barriers associated with stigma and geographic inaccessibility (20). Implementing similar models in India, particularly in rural and underserved areas, could significantly improve transgender individuals' access to gender-affirming mental health support. Nonetheless, significant obstacles exist, including inadequate infrastructure in rural clinics, low digital literacy among marginalized transgender populations, unaffordable costs of smartphones and data packages, resistance from clinicians unfamiliar with digital tools, and persistent policy inertia that collectively constrain the scalable implementation of AI-driven and telehealth interventions.

To ensure sustainable improvements in transgender healthcare, it is imperative to advocate for mandatory transgender-inclusive training in medical and psychiatric education. Medical and psychiatric curricula must be restructured to include transgender-specific modules on trauma-informed care, pain management, and gender diversity. Lifelong learning—via CPD modules and virtual platforms—should ensure practitioners maintain competency in delivering affirming, evidence-based care. Continuous professional development (CPD) modules, virtual learning platforms, and certification programs in gender-affirming care should be mandatory for practicing healthcare professionals to ensure up-to-date clinical competence. However, widespread adoption of such reforms faces significant barriers, including entrenched medical conservatism, bureaucratic inertia, and resistance from policymakers unwilling to challenge existing healthcare structures. Many institutions continue to view gender-affirming care as peripheral rather than integral to medical training, further stalling necessary reforms.

Furthermore, establishing legal and institutional frameworks to support AI-driven and telehealth interventions is critical. Policies must be introduced to regulate digital health platforms while ensuring accessibility, affordability, and privacy protections for transgender individuals seeking remote mental health support. However, the lack of political will and resistance to technological advancements in healthcare further complicate these efforts. Ensuring seamless integration of AI and telehealth interventions requires multi-stakeholder collaboration, where policymakers, healthcare providers, and LGBTQ+ advocacy groups work collectively to establish clear regulatory guidelines, data privacy protections, and culturally competent AI models that reflect the needs of the transgender community. Besides, increased funding and research grants dedicated to transgender healthcare studies in India must be prioritized. A robust investment in transgender-inclusive research will facilitate the development of targeted interventions, enabling evidence-based policy reforms. Yet, funding constraints and limited governmental interest in LGBTQ+ health research present critical challenges. To overcome these barriers, advocacy groups, academic institutions, and healthcare organizations must form strategic alliances to push for dedicated research grants and institutional funding mechanisms. Without financial commitment from public and private sectors, transgender healthcare will continue to be marginalized, and efforts to improve pain and mental health management will remain superficial.

Addressing these systemic failures requires an actionable roadmap where medical institutions, policymakers, and LGBTQ+ advocacy groups collaborate to dismantle discriminatory structures within healthcare. The first step is mandating transgender-inclusive education in all institutions for behavioral and health education, ensuring that future healthcare providers are equipped to deliver affirming care. Simultaneously, legislative interventions must enforce legal protections for AI-driven and telehealth solutions while funding allocations must be increased to support LGBTQ+ health research. Additionally, it is essential to acknowledge the pivotal role of community-driven, transgender-led organizations in addressing mental health and pain management, particularly in areas where institutional healthcare remains inaccessible. These grassroots networks bridge systemic gaps through peer counseling, advocacy, and the provision of culturally competent care. Strengthening partnerships between institutional healthcare systems and such community initiatives can facilitate more inclusive, localized, and accessible healthcare solutions. Without these fundamental shifts, transgender individuals will continue to face systemic neglect, denied the right to appropriate, evidence-based, and gender-affirming healthcare.

This letter calls for an immediate and systemic reformation of transgender pain and mental health management research in India. Establishing rigorous, evidence-based, and culturally competent clinical protocols is a medical and ethical necessity. Researchers, policymakers, and healthcare professionals must collaborate to dismantle the structural deficiencies that have historically excluded transgender individuals from receiving adequate pain and psychiatric care. Incremental reforms will be insufficient—comprehensive and immediate action is required to address the systemic inequities embedded within India's healthcare system. A failure to act will further entrench disparities, denying transgender individuals access to equitable and affirming healthcare and compromising their fundamental right to physical and psychological wellbeing. While this article draws upon existing research and global policy models, it does not present new empirical data. As an opinion-based synthesis, its primary contribution lies in critically framing neglected questions and advancing an urgent research and policy agenda for transgender pain and mental health equity. As an opinion-based synthesis, this article seeks to catalyze ethically grounded, empirically rich research that places transgender pain and resilience at the center of health policy discourse. This article calls on the research community, policy institutions, and funding bodies to recognize the epistemic urgency of transgender health inequities in India and to respond through rigorous, interdisciplinary, and community-partnered inquiry.
