# Supplementary material for: Unseen suffering: the urgent need for gender-affirming pain and mental health management for transgender individuals in India
Source: Front Public Health. 2025 Apr 29;13:1594703. doi: 10.3389/fpubh.2025.1594703 (PMC12069335; doi:10.3389/fpubh.2025.1594703)
Supplement: Supplementary file 1 [file Data_Sheet_1.docx]

**Supplementary Document: Clarification of Key Technical Terms**

This clarification note is intended to support interdisciplinary readers and reviewers by providing accessible definitions for key technical and conceptual terms used in the article *“Unseen Suffering: The Urgent Need for Gender-Affirming Pain and Mental Health Management for Transgender Individuals in India.”* These terms have been employed in the context of mental health, gender studies, digital health, and pain management.

**1. Systemic Health Disparities**

**Definition**: Health inequalities that arise from institutional structures and social systems rather than individual behavior. These disparities are sustained by policies, practices, and norms that create unequal access to healthcare services across populations.

**2. Neurobiological**

**Definition**: Pertaining to the biological mechanisms of the nervous system, including the brain, spinal cord, and neural pathways. In this context, the term relates to how gender-affirming hormone therapies influence neurological processes related to pain perception and mental health.

**3. Trauma-Informed Approaches**

**Definition**: Clinical and therapeutic strategies that recognize the prevalence and impact of trauma—particularly medical, social, or familial—on marginalized individuals. Such approaches emphasize safety, trust, empowerment, and cultural sensitivity in healthcare delivery.

**4. Intersectional Methodologies**

**Definition**: Research frameworks that explore how overlapping identities—such as caste, gender, geographic location, age, disability, and class—interact to produce compounded experiences of marginalization or privilege. These methodologies are especially crucial when studying health inequities in diverse, stratified societies.

**5. Gender Dysphoria / Experienced Gender**

**Definition**: Gender dysphoria refers to the distress that may accompany a discordance between an individual's experienced or expressed gender and the sex assigned at birth. The term “experienced gender” is increasingly used in affirming language to describe an individual’s authentic gender identity, without pathologization.

**6. Cognitive-Behavioral Therapy (CBT)**

**Definition**: A structured, evidence-based psychotherapeutic intervention aimed at altering dysfunctional thoughts and behaviors. CBT has been validated for the treatment of anxiety, depression, and chronic pain, and is adaptable for use in gender-affirming mental healthcare settings.

**7. Mixed-Methods Research**

**Definition**: A research design that integrates both qualitative (e.g., interviews, ethnography) and quantitative (e.g., surveys, longitudinal tracking) approaches to gain a more comprehensive understanding of complex social and health phenomena.

**8. Participatory Ethics**

**Definition**: An ethical approach to research that involves active collaboration with affected communities in defining research goals, processes, and dissemination. It emphasizes co-production of knowledge, reflexivity, and community empowerment.

**9. Digital Mental Health Tools / AI-Driven Models**

**Definition**: Technology-enabled platforms—including mobile apps, telehealth interfaces, and artificial intelligence (AI)-based chatbots—used to deliver mental health support. In this article, AI tools refer to systems capable of offering predictive diagnostics, therapeutic interactions, or administrative triaging tailored for marginalized populations.

**10. Telehealth / mHealth**

**Definition**: Telehealth refers to the provision of healthcare services remotely via telecommunications technologies. mHealth (mobile health) specifically uses mobile devices (e.g., smartphones, tablets) to deliver health services and information. These tools are increasingly important for reaching geographically isolated or socially marginalized communities.

**11. Continuous Professional Development (CPD)**

**Definition**: Ongoing education and training that healthcare professionals undertake to maintain and enhance their clinical competencies. In the context of this article, CPD includes learning modules on gender diversity, trauma-informed care, and affirming mental health practices.

**Note to Readers**: The inclusion of these terminologies reflects the interdisciplinary nature of the article, drawing on frameworks from psychiatry, public health, psychiatric social work, digital innovation, gender studies, and critical social theory. All terms have been operationalized to maintain fidelity to their disciplinary origins while being accessible to a broad readership.

For any additional clarification or terminological guidance, please contact the corresponding author.
